# Immunomodulatory Therapies for COVID-19 in Solid Organ Transplant Recipients

**DOI:** 10.1007/s40472-020-00306-x

**Published:** 2020-10-23

**Authors:** Mario Fernández-Ruiz, José María Aguado

**Affiliations:** 1 Unit of Infectious Diseases, Hospital Universitario “12 de Octubre”, Instituto de Investigación Sanitaria Hospital “12 de Octubre” (imas12), Centro de Actividades Ambulatorias, 2ª planta, bloque D. Avda. de Córdoba, s/n, 28041 Madrid, Spain; 2 Department of Medicine, School of Medicine, Universidad Complutense, Madrid, Spain

**Keywords:** COVID-19, Solid organ transplantation, Immunomodulatory therapy, Tocilizumab, Canakinumab, Colchicine

## Abstract

**Purpose of Review:**

Severe coronavirus disease 2019 (COVID-19) is characterized by the development of a deleterious hyperinflammatory response, in which the pleiotropic cytokine interleukin (IL)-6 plays a pivotal role. The administration of immunomodulatory therapies has been proposed to revert the tissue damage induced by COVID-19-related cytokine release syndrome (CRS). The present review summarizes the biological rationale and available clinical experience with this therapeutic strategy in the specific scenario solid organ transplantation (SOT).

**Recent Findings:**

A number of case reports, case series, and non-controlled cohort studies have assessed the efficacy and safety of the anti-IL-6-receptor monoclonal tocilizumab in SOT (namely kidney transplantation) recipients with COVID-19 pneumonia and CRS. Although the heterogeneity in patient management and the lack of a control group limit the interpretation of these results, tocilizumab therapy appears to provide some clinical benefit in post-transplant COVID-19 and to be reasonably safe in terms of bacterial superinfection. A large randomized clinical trial (RCT) has shown survival benefit with adjuvant corticosteroids in non-transplant patients, but supporting evidence is scarce for SOT recipients and confounded by the variable adjustment of baseline immunosuppression. Anecdotal experiences have been reported with the use of the anti-IL-1 agent anakinra and the NLRP3 inflammasome inhibitor colchicine in this population.

**Summary:**

Immunomodulation has emerged as a promising option for SOT recipients with COVID-19-related CRS, with available experience mainly restricted to the anti-IL-6 agent tocilizumab. However, the supporting evidence is scarce and of low quality. In the absence of RCT, observational studies including well-matched control groups should be designed in future.

## Introduction

Since the reporting in December 2019 of a cluster of acute respiratory illness of unknown origin linked to a seafood wholesale market in the city of Wuhan, Hubei, China [1], the coronavirus disease 2019 (COVID-19) pandemic is posing an unprecedented challenge to health care systems and transplant programs worldwide. One of the pathogenic hallmarks of infection due to severe acute respiratory syndrome (SARS) coronavirus 2 (SARS-CoV-2) is the development of an exuberant (and ultimately deleterious) inflammatory response orchestrated by the host immune system [2]. Indeed, the widespread tissue injury may result in the development of multiorgan failure (MOF) and acute respiratory distress syndrome (ARDS), which underlies most fatal COVID-19 cases [3].

The cytokine release syndrome (CRS), often termed as “cytokine storm,” is an unregulated cascade of auto-amplifying cytokine production triggered by different events, such as immune-related conditions, cancer, sepsis, or viral infections like influenza [4, 5]. The recent advent of chimeric antigen receptor (CAR) T cell therapy or bi-specific T cell engagers (i.e., blinatumomab) has been also accompanied by the occurrence of potentially life-threatening CRS in a subset of patients with hematological malignancies [6]. Similarly to that observed in SARS-CoV and Middle East respiratory syndrome (MERS)-CoV infections, a variety of both pro- and anti-inflammatory cytokines have been shown to be elevated in patients with severe COVID-19. Plasma levels of some of these mediators, such as IP-10 (interferon (IFN)-γ-inducible protein 10) and MCP-3 (monocyte-chemotactic protein 3), are highly correlated with disease severity and may predict progression to ARDS [7]. However, interleukin (IL)-6—a pleiotropic cytokine released by T cells, endothelial cells, fibroblasts, macrophages, and monocytes in acute and chronic inflammatory phases—appears to play a central role in the pathogenesis of COVID-19-related CRS. Severely ill patients infected with SARS-CoV-2 show low absolute lymphocyte counts and elevated C-reactive protein (CRP) levels. In contrast to the CRS associated with other conditions (in which splenomegaly and lymphadenopathy are common), autopsy findings have revealed spleen and lymph node atrophy in patients with COVID-19 [4]. It has been shown that SARS-CoV-2 selectively induces macrophages to produce IL-6, blocking lymphopoiesis and promoting excessive neutrophil recruitment in tissues [2]. When acting on hepatocytes, IL-6 induces the synthesis of a wide range of acute-phase reactants, including CRP. In accordance with these findings, since the earliest phases of the pandemic, the amount of circulating IL-6 has been consistently correlated with poor outcomes, including all-cause mortality [8, 9], or the requirement for invasive mechanical ventilation (IMV) [10]. On the other hand, by activating the NLRP3 inflammasome, SARS-CoV-2 also induces the synthesis of IL-1β and IL-18, which lead in turn to the release of IL-6 and IFN-γ, respectively [11].

Unremitting fever, ARDS, cytopenias, hyperferritinemia, elevated plasma cytokine levels, liver and renal failure, and coagulation abnormalities are common features of COVID-19-related CRS that resemble those observed in immune-mediated conditions such as secondary hemophagocytic lymphohistiocytosis or in the setting of CAR T cell therapy. Therefore, the existence of overlapping pathogenic mechanisms and clinical manifestations has supported the use of immunomodulatory therapies to counteract the cytokine-induced tissue damage in the lung and other organs triggered by SARS-CoV-2. This approach is based on the repurposing of agents previously approved for autoimmune and inflammatory conditions, including those targeting cytokines such as IL-6 (tocilizumab (TCZ), sarilumab) or IL-1β (anakinra, canakinumab), receptor-associated kinases such as the Janus kinase (JAK) family (baricitinib), or the NLRP3 inflammasome (colchicine) [12].

Although it was first hypothesized that the long-term immunosuppressive therapy received by solid organ transplant (SOT) recipients would minimize to some extent the hyperinflammatory state associated with COVID-19, thus resulting in better patient outcomes [13, 14], accumulating evidence suggests that the severity of SARS-CoV-2 infection in this population is at least comparable to that observed in non-transplant patients with similar age and comorbidities. The lack of effective antiviral drugs against coronaviruses and the risk of alloreactivity resulting from the rapid withdrawal of immunosuppression make immunomodulatory therapies especially appealing as therapeutic approach in post-transplant COVID-19. Nevertheless, potential concerns due to the increased risk of bacterial or opportunistic infections should be also considered. The present review is aimed at overviewing the available experience on the effectiveness and safety of immunomodulatory therapies for the treatment of COVID-19 in SOT recipients, with focus on IL-6-targeted agents in view of the more extensive evidence as compared to other therapeutic groups.

## IL-6 Blockade for COVID-19-Related CRS

### Evidence in the Non-transplant Population

Tocilizumab is a humanized monoclonal antibody that binds both the soluble and membrane-bound forms of the IL-6 receptor (IL-6R). This biological agent was initially approved by US and European regulatory agencies for the treatment of rheumatoid arthritis and juvenile idiopathic arthritis [15] and, more recently, for patients with CRS associated with CAR T cell therapy [16] and giant cell arteritis [17] (the latter indication only granted by the Food and Drug Administration). The dramatic need of treatment options for SARS-CoV-2 infection, the instrumental role played by IL-6 in the pathogenesis of COVID-19-associated CRS, and the well-known safety profile of the agent [18, 19] prompted the off-label use of TCZ early in the course of the pandemic. The pivotal study by Xu et al. analyzed the retrospective experience with 21 severe or critically ill COVID-19 patients (including three undergoing non-invasive ventilation or IMV), in which body temperature, pulse oximetry oxygen saturation (SpO_2_), serum CPR levels, and lymphocyte count returned to normal ranges within the first 5 days from the administration of one or two intravenous (IV) doses of TCZ (4–8 mg/kg body weight). Three quarters of the patients experienced a decrease in oxygen requirements, with overall rates of clinical and radiological response above 90.0%. The authors reported no serious adverse events or the emergence of bacterial, fungal, or viral superinfections during the course of treatment [20]. These promising preliminary results were followed by a growing number of case series and non-controlled cohort studies, mainly single-center and retrospective in design [21–29]. These studies, however, largely differed in the timing of dosing in relation to infection and in inclusion criteria (i.e., severity of respiratory failure or IL-6 threshold for the initiation of treatment), background therapies, length of follow-up, and analyzed endpoints (from clinical improvement or requirements for intensive care unit (ICU) admission and IMV to all-cause mortality). In a large retrospective study that recruited 544 patients from three Italian centers, Guaraldi et al. found that the administration of either IV or subcutaneous (SC) TCZ was associated with a reduction in the risk of IMV or death as compared to standard care alone, with a more evident effect in the subgroup with a baseline partial pressure of arterial oxygen to fraction of inspired oxygen (PaO_2_/FiO_2_) ratio lower than 150 [30]. Further case-control studies, however, have yielded conflicting results, with some of them suggesting clinical benefits in terms of decreased mortality or IMV [31, 32], whereas others failed to demonstrate differences between TCZ-treated patients and those receiving standard of care only [33, 34]. While waiting for results from ongoing randomized clinical trials (RCTs), and on the basis of available studies of low-to-moderate methodological quality, there remain uncertainties regarding the actual benefit derived from the use of TCZ as immunomodulatory therapy in COVID-19, as highlighted by a recent meta-analysis (pooling data from 7 observational studies and 592 patients) that found no significant differences in any of the analyzed outcomes [35].

Sarilumab, an anti-IL-6 monoclonal antibody, has been also investigated in patients with severe COVID-19 pneumonia and associated CRS although the available experience is scarce [33, 36].

### Clinical Experience with TCZ in SOT Recipients

Likely reflecting the increasing experience in the non-transplant population (and the promising results initially reported), immunomodulatory therapy based on TCZ has emerged as one of the preferred approaches in SOT recipients with COVID-19-related CRS. In a recent systematic review that pooled data from 12 case series reported through June 2020 totaling 204 kidney transplant (KT) recipients, TCZ was used in 14.3% patients with available data. All-cause mortality in the overall cohort was 21.1%, and 19.7% recipients required IMV [37]. Consistent with this figure, 13.4% of 144 KT recipients in the international TANGO consortium [38] and 13.1% of 482 SOT recipients reported within a US multicenter prospective cohort [39•] received TCZ or sarilumab. A nationwide registry maintained by the Spanish National Transplant Organization reported the administration of TCZ in 105 out of 713 (14.7%) recipients with COVID-19 across 82 different centers (as compared to only 1.1% that received anakinra). Of note, there were large differences in the proportion of patients treated with TCZ according to the transplant type (from 47.4% of lung or 22.2% of heart recipients to 7.7% of liver recipients), which would reflect disparities in disease severity [40].

First case reports describing the off-label use of TCZ in SOT recipients usually showed favorable responses, raising suspicions of potential publication bias [41–48]. Fontana et al. observed a rapid clinical improvement following a single SC administration of TCZ (324 mg) in a 61-year-old KT recipient with bilateral interstitial pneumonia and respiratory failure, but not yet progressing to ARDS. The authors discussed the potential benefit expected from the early initiation of therapy, before overt lung damage ensued [41]. A similarly favorable outcome was reported in a liver transplant (LT) recipient with increasing levels of inflammatory markers that never developed respiratory failure [42]. A single IV infusion of TCZ (8 mg/kg body weight) also resulted in the rapid decrease of CPR levels and oxygen requirements over the next 48 h in a 69-year-old KT recipient with markedly elevated IL-6 levels at baseline (431 pg/mL) [44].

Beyond these preliminary reports, the first case series rapidly ensued (summarized in Table 1). All of them are limited, however, by low sample sizes and the lack of a control group not treated with TCZ. Therapeutic management, in addition, was heterogeneous both across and within studies, with most patients undergoing reduction of immunosuppression (usually in form of discontinuation of antimetabolites and/or dose reduction of the calcineurin inhibitor). In addition, some of them also received corticosteroids pulses or polyclonal intravenous immunoglobulins (IVIg) prior to or at the time of the first infusion of TCZ. Since candidates to receive immunomodulatory therapies are typically those with more severe clinical courses, the majority of the cases had been also treated with agents with presumed activity against SARS-CoV-2, such as lopinavir/ritonavir, hydroxychloroquine, or azithromycin. Although subsequent RCTs have not consistently demonstrated any clinical benefit for these agents [49, 50], the potential occurrence of adverse events and drug-drug interactions with immunosuppressive drugs is likely to confound the interpretation of the findings.

**Table 1:** Review of studies assessing the use of TCZ as immunomodulatory therapy in SOT recipients with COVID-19 pneumonia

First author (ref)	Patients	Inclusion criteria	TCZ dosing regimen	Time interval from symptoms onset	Clinical outcomes^a^
Pereira [51]	14 SOT recipients	Not provided in detail (rapid clinical decompensation due to CRS)	One single dose (*n* = 9), two doses (*n* = 4), three doses (*n* = 1)	Not provided	Overall mortality: 21.4% ICU admission: 42.3% Discharge: 14.3%
Bossini [53]	8 KT recipients	Clinical deterioration after ≥ 7 days from symptoms onset or no fever for > 72 h, escalating oxygen requirements, radiological progression, and no signs of bacterial infection	Up to two IV doses (8 mg/kg, maximum per infusion 800 mg) at an interval of 12–24 h associated with dexamethasone	Not provided	Overall mortality: 37.5% Clinical improvement: 62.5% Discharge: 37.5%
Mella [54].	6 KT recipients	SpO_2_ < 93% on room air or PaO_2_/FiO_2_ ratio < 300, and increased CRP and/or IL-6 levels (× 10 lowest reference range)	Two IV doses (8 mg/kg)	Not provided	Overall mortality: 66.7% Bacterial infection: 16.7%
Pérez-Saez [56]	80 KT recipients	Increased IL-6 levels (> 40 pg/mL), increased levels of other inflammatory markers (CRP, D-dimer, ferritin, or LDH), and/or ARDS	One single IV dose (8 mg/kg) (*n* = 64), ≥ 2 doses (*n* = 16)	Median: 10 days (IQR: 7–15)	Overall mortality: 32.5% ICU admission: 30.0% IMV: 25.0% Acute kidney injury: 45.0% Acute rejection: 1.3% Bacterial infection: 7.5%
Trujillo [55]	10 KT recipients	One or more of the following: (a) respiratory frequency > 30 bpm and/or SpO_2_ < 92%; (b) escalating oxygen requirements in the prior 24–48 h; (c) CRP levels ≥ 10 mg/dL; (d) IL-6 levels > 40 pg/mL; (e) D-dimer levels > 1500 ng/mL	One single (400 mg if body weight < 75 kg or 600 mg if weight ≥ 75 kg) IV dose	Mean: 13 ± 4 days	Overall mortality: 30.0% Clinical improvement: 70.0% Bacterial infection: 0.0% Invasive aspergillosis: 10.0%

^a^Patient outcomes at the time of reporting

*ARDS*, acute respiratory distress syndrome; *CRP*, C-reactive protein; *CRS*, cytoquine release syndrome; *ICU*, intensive care unit; *IL*-*6*, interleukin 6; *IMV*, invasive mechanical ventilation; *IQR*, interquartile range; *IV*, intravenous; *KT*, kidney transplantation; *LDH*, lactic acid dehydrogenase; *PaO*_*2*_*/FiO*_*2*_, partial pressure of arterial oxygen to fraction of inspired oxygen; *SOT*, solid organ transplantation; *SpO*_*2*_, pulse oximetry oxygen saturation

Within a larger cohort of SOT recipients recruited during the initial weeks of the pandemic in two centers in New York City, Pereira et al. reported the outcomes for 14 patients treated with IV TCZ (mainly as a single-dose regimen) after experiencing a rapid clinical deterioration. In fact, five of them (35.7%) received the first TCZ dose once IMV had been initiated. At the time of reporting, three patients (21.4%) had died, four (28.6%) remained in the ICU, and only two (14.3%) had been discharged. Unfortunately, no further details on the clinical or laboratory evolution were provided [51]. A case series of 20 KT recipients from three transplant centers in Brescia (Italy) during the first weeks of the pandemic included 6 patients treated with up to 2 IV infusions of TCZ in addition to dexamethasone (20 mg daily for 5 days followed by 10 mg daily for the next 5 days). Two patients (33.3%) died, three patients remained hospitalized, and only one of them was discharged [52]. The same group has enlarged this multicenter cohort with updated follow-up, reporting an overall mortality of 37.5% among 8 KT recipients with ARDS treated with TCZ and dexamethasone [53]. In a small single-center case series, Mella et al. reported that 4 out of 6 KT recipients died after a mean interval of 9.75 days from the first TCZ dose. Recovery of the lymphocyte count was observed in the two surviving recipients [54].

We have analyzed the first 10 KT recipients treated with TCZ for SARS-CoV-2 infection at our center (Hospital Universitario “12 de Octubre,” Madrid, Spain) between March 26 and April 18. Four of them were receiving high-flow supplemental oxygen (FiO_2r_ ≥ 40%) or non-invasive mechanical ventilation at the time of therapy. All the patients exhibited a rapid decrease in serum CRP levels over the first 48 h from the administration of a single IV dose of TCZ (400 mg if body weight < 75 kg or 600 mg if ≥ 75 kg), with no differences in lactic acid dehydrogenase (LDH) or absolute lymphocyte counts. After a median follow-up of 16 days, 7 patients (70%) experienced clinical improvement with progressive reduction in oxygen requirements and were finally discharged, whereas the remaining 3 recipients eventually died. There was a non-significant trend towards a more delayed administration of TCZ from the initiation of symptoms among patients with poor outcomes compared to those that responded to treatment (median of 11 and 9 days, respectively) [55].

The largest study to date on the effectiveness and safety of TCZ for post-transplant COVID-19 included 80 KT recipients from 29 transplant centers at Spain [56••]. Although the criteria for initiation of therapy differed across institutions, all patients showed increased levels of IL-6 (most often > 40 pg/mL) and other inflammatory biomarkers (such as CRP, D-dimer, ferritin, or LDH), and/or rapidly progressive ARDS (usually defined by a PaO_2_/FiO_2_ ratio < 300). The median interval from the onset of symptoms to the administration of TCZ was 10 days, and only 20.0% of recipients received a repeated dose. Most patients were also treated with hydroxychloroquine (98.8%) and antibiotics (76.3%), as well as corticosteroid boluses (80.0%). Only serum CRP levels markedly decreased early after the administration of TCZ, in contrast with other inflammatory markers such as ferritin that actually increased over time. The case fatality rate was 32.5%, with patient age > 60 years (hazard ratio (HR): 3.12; 95% confidence interval (CI): 1.05–9.26) and high CRP levels after TCZ administration (HR (per 1 mg/dL increase): 1.01; 95% CI: 0.004–1.024) being identified as independent predictors of all-cause mortality. The previous or concomitant use of corticosteroids, SC IFN-β, and anakinra was more common among non-survivors, as was ICU admission at the time of therapy, although the lack of significant association after multivariate adjustment for any of these variables would suggest confounding by indication [56••]. Nevertheless, the deleterious impact of IFN-β on the outcome of TCZ-treated patients has been also reported for the non-transplant population [25].

Finally, data from an international consortium found no crude differences in all-cause mortality between KT recipients treated or not treated with TCZ (42.1% versus 30.4%, respectively), although the number of treated patients was low (*n* = 19) and no adjustment for disease severity was performed [38].

### Safety Concerns

A large amount of data from non-transplant patients with rheumatoid arthritis and other rheumatic disorders indicates that long-term therapy with TCZ may increase the risk of opportunistic and serious bacterial infection, with pneumonia, urinary tract infection, and cellulitis as the most common complications [57]. In addition, alteration of liver function tests, elevation of total and low-density lipoprotein cholesterol levels, and lower gastrointestinal perforation have been identified as TCZ-associated adverse events [58]. It should be considered, nevertheless, that the initiation of TCZ therapy for these indications is usually preceded by a baseline evaluation aimed at minimizing the risk of reactivation of latent infections (i.e., tuberculin skin test), which is not feasible in the COVID-19 setting due to the dramatic course of the infection and the narrow window of opportunity for immunomodulation.

Previous experience with the use of TCZ for the treatment of antibody-mediated rejection (AMR) suggests that this agent is relatively safe in the SOT population. In a cohort of 36 KT recipients with chronic AMR that had failed previous treatment with IVIg and rituximab, salvage therapy with monthly TCZ was associated with an overall rate of infection of 36.1%. None of these episodes (primarily bacterial infection) required hospitalization or prompted the discontinuation of therapy. In addition, eight patients developed hypogammaglobulinemia during therapy, although the role of B cell depletion due to previous treatment with rituximab should be considered [59]. Similar results were reported from a single-center study including 15 KT recipients with chronic AMR that received TCZ as first-line therapy, with a cumulative incidence of bacterial infection of 33.3% after a median follow-up of 20.7. Most of the episodes were urinary tract infections, and again none of them led to hospitalization or therapy discontinuation. One patient developed encephalitis of unknown origin 3 months after the initiation of TCZ, with complete recovery following the temporary discontinuation of treatment [60]. The occurrence of cytomegalovirus esophagitis and hypersensitivity reaction was reported in one case each out of seven KT recipients treated with monthly TCZ for a median of 4 months for acute active AMR [61]. Of note, all these studies evaluated the safety of TCZ over prolonged courses of therapy, which is in contrast with the one- to three-dose regimen usually administered for COVID-19.

The rates of bacterial or fungal superinfection complicating TCZ therapy in non-transplant patients with COVID-19 are quite variable across studies, ranging from less than 5% [22, 28] to 15–30% [29, 30] or above 50% [62•]. Such large discrepancies are likely explained by differences in study definitions (e.g., inclusion of episodes with microbiological documentation only versus those empirically treated), magnitude of diagnostic efforts, and baseline clinical severity (with higher incidence of subsequent infection among patients mechanically ventilated at the time of administration of TCZ [62•]). In addition, the causal attribution of IL-6 blockade is not straightforward since COVID-19 patients already face an increased risk of developing infectious complications, in particular those with prolonged ICU stays. The sequential of concomitant use of high-dose corticosteroids as immunomodulatory strategy may also contribute to this complication. In fact, some case-control studies report no differences in the rates of bacteremia or other adverse events between patients receiving TCZ or sarilumab compared to the standard of care group [33, 34]. Of note, statistical power may have been inadequate to reveal important safety signals.

These limitations are particularly evident when addressing the safety of TCZ-based immunomodulation for post-transplant COVID-19. No episodes of bacterial or fungal superinfection were observed in the published case reports [43–45, 47], although antibiotic prophylaxis was common and only short-term follow-up was described in most of them. None of the 10 first KT recipients in our institutional cohort developed bacterial or viral infections, and one of them was diagnosed with invasive pulmonary aspergillosis at day 10 from the receipt of a single dose of TCZ [55]. The incidence of bacterial pneumonia in the Spanish multicenter study discussed above was 7.5%, and all the 6 KT recipients diagnosed with this complication had also received corticosteroid pulses. No information on other adverse events, such as gastrointestinal perforation or dyslipidemia, was provided [56•]. One case of septic shock due to *Klebsiella pneumoniae* with fatal outcome was observed in a case series of 6 KT recipients [54]. Any conclusion on the safety profile of TCZ, however, should be viewed with caution due to the lack of adequately powered studies in the SOT population with detailed follow-up data.

## Other Immunomodulatory Therapies

### Anti-IL-1 Agents

Anakinra is a recombinant IL-1 receptor antagonist (IL-1Ra) that differs from its native counterpart in the presence of an extra methionine residue at the amino terminal end. By mirroring the mode of action of the endogenous form, anakinra inhibits the binding of IL-1α and IL-1β to IL-1R, and it has been approved for the treatment of rheumatoid arthritis, adult-onset Still’s disease, and certain periodic fever syndromes (such as familial Mediterranean fever). To date, available experience with the use of anakinra as immunomodulatory therapy in SARS-CoV-2 infection is mainly restricted to case series and small uncontrolled studies [63–67]. The use of high-dose (5 mg/kg twice daily) IV anakinra, but not low-dose (100 mg twice daily) SC regimen, was associated with higher rates of clinical improvement and patient survival as compared to historical controls [63•]. A small retrospective study performed in three French centers compared 22 patients with COVID-19 and associated ARDS who received either standard of care treatment alone (10 patients) or combined with IV anakinra (12 patients). The dosing regimen was 300 mg daily for 5 days, with subsequent tapering (200 mg daily for 2 days and then 100 mg for one further day). Clinical improvement by day 5 was more common among patients that received anakinra, who also had a more rapid resolution of fever and normalization of CRP levels as compared to the control group [11]. In a non-controlled, single-center cohort, 7 out of 11 patients treated with SC anakinra (100 mg every 6 h with slow tapering for a maximum of 20 days) early after the onset of hypoxemic respiratory failure remained free from IMV and were eventually discharged home. The presence of high ferritin levels (> 1000 ng/mL)—a hallmark of CRS driven by IL-1—was an entry criterion [68]. There are no data specifically assessing the effectiveness and safety of anakinra among SOT recipients with COVID-19. In the Spanish National Transplant Organization registry, only 8 out of 713 patients (1.1%) received anakinra (all of them KT recipients), although no disaggregated data was available for this small subgroup [40]. Consistent with this limited experience available for IL-1 blockade in SOT, none of the 144 KT recipients reported by the international TANGO consortium was treated with anakinra [38].

### Corticosteroids

Despite the lack of data demonstrating a net clinical benefit in patients with SARS-CoV or MERS-CoV infection [69], the administration of corticosteroids rapidly emerged as a therapeutic option to abrogate CRS triggered by SARS-CoV-2 [70]. In addition to uncertainties on the optimal dosing and timing, concerns were also raised on the potential deleterious impact on the host ability for pathogen clearance. Indeed, a recent meta-analysis (mostly comprising observational studies) concluded that corticosteroid therapy was associated with significantly higher mortality and incidence of nosocomial infection in patients with severe influenza and ARDS [71]. In addition, more prolonged viral shedding was observed for patients hospitalized with influenza A (H7N9) pneumonia treated with high-dose corticosteroids (80-mg methylprednisolone daily or equivalent) as compared to the control group [72].

A quasi-experimental, pre-post-study concluded that the early use of short-course methylprednisolone (0.5 to 1 mg/kg daily for 3 days) in adult patients with moderate to severe COVID-19 was associated with a lower incidence of the composite endpoint of ICU admission, new requirement for IMV, and mortality [73]. The most definitive evidence, however, on the utility of corticosteroids for immunomodulation in SARS-CoV-2 infection has been provided by a multicenter open-label RCT performed in the UK (the only one to date to demonstrate a survival benefit in COVID-19), in which patients were randomized to receive oral or IV dexamethasone (at a dose of 6 mg once daily) for up to 10 days or standard care alone. A significant (albeit modest) reduction in the primary outcome of 28-day mortality was preliminary reported for the dexamethasone group compared to the control group (22.9% versus 25.7%, respectively; rate ratio: 0.83; 95% CI: 0.75–0.93). This protective effect was more among patients receiving IMV at randomization (29.3% versus 41.4%), although no differences were observed for those not receiving oxygen support [74••].

Unfortunately, no studies have specifically assessed outcomes for SOT recipients with COVID-19 treated with corticosteroids. As much as two-thirds of the KT recipients in the TANGO registry received “increased corticosteroids,” with no further details on dosing or length of therapy and no differences among survivors and non-survivors [38]. In the Spanish multicenter study on TCZ, 80.0% of patients also received adjuvant corticosteroid therapy [56••]. Pereira et al. detailed patient outcomes for 16 SOT recipients treated with boluses, with all-cause mortality of 18.8% and 12.5% discharged home at the time of reporting [51]. In addition to the non-randomized design of these studies and the lack of details on the regimen administered (i.e., type of corticosteroid and relative potency, low-to-moderate daily courses versus high-dose pulses for few days), the analysis is hampered by the large heterogeneity observed in the adjustment of baseline immunosuppression. A literature review pooling data for 41 KT recipients described three separate basic schemes (no changes in immunosuppression, only antimetabolites were suspended, or only maintained on corticosteroids), which greatly differed according to the severity of SARS-CoV-2 infection, pointing to confounding by indication [75]. Consistent with this notion, as much as 25.7% of LT recipients with severe COVID-19 reported from a large Spanish multicenter study received corticosteroid boluses, compared to only 4.9% of those with mild or moderate infection [76•].

### Colchicine

The long-known anti-inflammatory effect of colchicine is mediated by different routes, including the inhibition of neutrophil chemotaxis and activation in response to vascular injury, blockade of NLRP3 inflammasome signaling, and impaired IL-1β production [77]. As discussed above, SARS-CoV-2 has been implied in the activation of NLRP3 inflammasome through the accessory protein viroporin E. On the basis of this rationale, and considering its acceptable safety profile and low price, colchicine has been proposed to be repurposed as anti-inflammatory therapy in patients with severe COVID-19 pneumonia. An open-label RCT found that patients treated with colchicine had lower rates of clinical deterioration as compared to those allocated to the control group, with no differences in peak CRP or cardiac troponin levels. The occurrence of diarrhea was more common with colchicine [78]. There is anecdotal experience with the use of colchicine as immunomodulatory therapy for COVID-19 in SOT. Gandolfini et al. reported the case of a 52-year-old KT recipient extensive bilateral ground-glass lung opacities and elevated IL-6 levels that received colchicine (1 mg on the first day, and 0.5 mg daily thereafter) due to the shortage of TCZ. Serum IL-6 rapidly decreased within the first 24 h from 108.2 to 36 pg/mL, with concurrent improvement in the respiratory status [79].

## Conclusions

The use of immunomodulatory therapies to prevent the development of ARDS and MOF induced by COVID-19-related CRS possesses both biological plausibility and promising clinical evidence. Nevertheless, all studies published to date in non-transplant patients are of low methodological quality and suffer from high risk of bias [35]. Therefore, any conclusion on the potential benefit derived from this approach should be considered as preliminary, pending on results from ongoing RCTs. Major questions remain regarding the optimal agent (IL-6 or IL-1 blockade with or without adjuvant corticosteroids), regimen (single-agent or sequential therapy against different immune targets), and timing, as well as on the potential effect on the capacity for viral clearance—a relevant concern due to the lack of effective antiviral drugs for SARS-CoV-2—and the risk of bacterial superinfection. Reflecting these knowledge gaps, the latest version of the Infectious Diseases Society of America (IDSA) guidelines on the treatment of COVID-19 has emphasized that TCZ should be only administered in the context of a clinical trial [80]. Unfortunately, the evidence available for the specific SOT population is even weaker, mostly restricted to TCZ and based on single case reports and underpowered non-comparative studies (Table 1). Data on effectiveness and safety coming from studies conducted in the general population are not necessarily applicable to the SOT setting. Although preliminary experiences suggest that IL-6 blockade is relatively safe in post-transplant COVID-19, the risk of potentially severe adverse events and the absence of conclusive evidence on the clinical benefit to be expected from TCZ therapy should be carefully balanced in the clinical decision-making process. Since the conducting of RCTs focused on SOT recipients with COVID-19 is unlikely due to logistical issues, observational multicenter studies with well-matched control groups not receiving immunomodulation will be highly welcomed.
